# Microreact: visualizing and sharing data for genomic epidemiology and phylogeography

**DOI:** 10.1099/mgen.0.000093

**Published:** 2016-11-30

**Authors:** Silvia Argimón, Khalil Abudahab, Richard J. E. Goater, Artemij Fedosejev, Jyothish Bhai, Corinna Glasner, Edward J. Feil, Matthew T. G. Holden, Corin A. Yeats, Hajo Grundmann, Brian G. Spratt, David M. Aanensen

**Affiliations:** ^1^​ The Centre for Genomic Pathogen Surveillance, Wellcome Genome Campus, Hinxton CB10 1SA, UK; ^2^​ Department of Infectious Disease Epidemiology, Imperial College London, London W2 1PG, UK; ^3^​ The Milner Centre for Evolution, Department of Biology and Biochemistry, University of Bath, Bath, UK; ^4^​ School of Medicine, University of St. Andrews, St. Andrew, UK; ^5^​ Department of Infection Prevention and Hospital Hygiene, University Medical Centre Freiburg, Freiburg, Germany

**Keywords:** Open data, phylogenomics, phylogeography, population genomics, trees

## Abstract

Visualization is frequently used to aid our interpretation of complex datasets. Within microbial genomics, visualizing the relationships between multiple genomes as a tree provides a framework onto which associated data (geographical, temporal, phenotypic and epidemiological) are added to generate hypotheses and to explore the dynamics of the system under investigation. Selected static images are then used within publications to highlight the key findings to a wider audience. However, these images are a very inadequate way of exploring and interpreting the richness of the data. There is, therefore, a need for flexible, interactive software that presents the population genomic outputs and associated data in a user-friendly manner for a wide range of end users, from trained bioinformaticians to front-line epidemiologists and health workers. Here, we present Microreact, a web application for the easy visualization of datasets consisting of any combination of trees, geographical, temporal and associated metadata. Data files can be uploaded to Microreact directly via the web browser or by linking to their location (e.g. from Google Drive/Dropbox or via API), and an integrated visualization via trees, maps, timelines and tables provides interactive querying of the data. The visualization can be shared as a permanent web link among collaborators, or embedded within publications to enable readers to explore and download the data. Microreact can act as an end point for any tool or bioinformatic pipeline that ultimately generates a tree, and provides a simple, yet powerful, visualization method that will aid research and discovery and the open sharing of datasets.

## Data Summary

1. Microreact is freely available (at http://microreact.org).
2. Microreact – Hierarchical and Geographical Analysis Tool.
3. Microreact allows you to upload, visualize and explore dendrograms (trees) linked to adata containing geographical locations.

## Impact Statement

Large genome sequencing projects are generating vast amounts of sequence data and related metadata, and bringing about great advances in the fields of microbial genomics and infectious-disease epidemiology. Tools to visualize, explore and share large genomic surveys are, however, lacking. We present Microreact, a web application that combines clustering, geographical and temporal data into an interactive visualization with trees, maps, timelines and tables. The dynamic nature of a Microreact visualization can better illustrate findings than the static tree figures commonly generated by bioinformatics pipelines. In addition, data files can be linked to Microreact from cloud servers, making it possible for all the users of a project to modify the data independently and to see the changes made by other users. Another key advance is that the Microreact visualization can be shared via a web link unique to each project, which extends the benefits of an interactive tool to collaborators and the research community. The Microreact web link can also be included into a publication, which democratizes the availability of data and the ability to explore it, in accordance with the open-access policies of many scientific journals.

## Introduction

A commonly used method for analysing large datasets consisting of many individual entities is to cluster data points and to represent relationships diagrammatically as a tree, in which closeness on the tree indicates relatedness according to the chosen measure of similarity. Relating allied data, for example spatial and temporal information, to the tree allows a deeper understanding of the system under investigation. Static trees summarising large datasets in this fashion are commonly used within the scientific literature to convey conclusions for a wide range of subjects, from human evolution (Hallast *et al.*, 2015) to the evolution of language (Currie *et al.*, 2013). However, static figures tend to only illustrate a broad view of a tree, or are chosen by authors to illustrate a particular aspect of a dataset; thus, precluding further interrogation of the data by the reader or the broader community.

This issue is particularly daunting within the field microbial genomics, where datasets and trees are exponentially increasing in size as the cost of whole-genome sequencing (WGS) decreases. In fact, microbial genomics has seen a clear shift from sequencing single representatives of a microbial species to sequencing large numbers of representatives from a population, providing data with the resolution to distinguish between highly similar genotypes. Within microbiology, WGS has been used to investigate the epidemiological and population dynamics of viral [e.g. Zika (Faria *et al.*, 2016) and Ebola (Quick *et al.*, 2016) viruses], bacterial [e.g. *Shigella dysenteriae* (Njamkepo *et al.*, 2016) and *Staphylococcus aureus* (Aanensen *et al.*, 2016)] and eukaryotic organisms [e.g. *Leishmania donovani* (Imamura *et al.*, 2016) and *Shistosoma mansoni* (Crellen *et al.*, 2016)]. The acquisition and analysis of sequence data in such studies follows a similar route. In brief, samples are collected and subjected to WGS using one of a number of sequencing platforms [e.g. Illumina, PacBio, Nanopore (Goodwin *et al.*, 2016)], followed by data processing using a series of bioinformatics steps (e.g. Page *et al.*, 2015; Vernikos *et al.*, 2015; Xiao *et al.*, 2015) that aim to determine the genetic differences among samples and infer evolutionary relationships between them. The combination of different sequencing platforms and bioinformatics methods yields a bewildering number of possible pipelines for microbial genomics. However, by far the most common final output is a tree that represents relationships between isolates. Data (e.g. geographical origins and dates of isolation of samples) are then co-analysed with the tree topology to further explore and interpret the data. These tree–metadata combinations are almost uniformly presented as static images even when they represent thousands of samples (Wong *et al.*, 2015), with only partial aspects of the data, either spatial or epidemiological, represented in the image.

With the dramatic increase in sequence data generated by WGS, there is an associated need for visualization platforms that not only provide a dynamic experience of data exploration (as opposed to static figures), but also facilitate collaborative, interdisciplinary investigations. Importantly, with the ongoing effor﻿ts to introduce WGS as a method for routine epidemiological investigation and surveillance of pathogens, there is a compelling need for generic, user-friendly methods to rapidly visualize and present the output of sequence data pipelines to a range of audiences with different expertise for collective interpretation (e.g. the research community, epidemiologists, bioinformaticians and public-health workers).

Here, we describe Microreact (www.microreact.org), a generic web application for the provision of interactive data visualizations, consisting of trees, maps and timelines linked to a user-defined array of metadata. While Microreact is applicable to a broad range of data types, we outline its generic functionality and illustrate it with examples from the field of microbial genomics. The input to Microreact is a data file that can contain a combination of textual metadata, locations and dates, along with an optional tree file. The output is a permanent web application consisting of an interactive tree, map, timeline and table. These four sections are linked, and subsets of data can be selected, filtered and queried simultaneously on all of them. The Microreact instance can be shared for collaboration or publication via a unique web link; thus, enabling collaborators or the research community to further explore and download the dataset(s). A Microreact instance produced for an individual project is henceforth called a ‘Microreact'.

Here, we give an overview of how to create a Microreact, including input and output specifications, using screenshots and links to the application. We then apply this to two published datasets, illustrating the increased interactivity and utility of the method for further community use. Finally, we describe a method for linking Microreact to dynamic datasets that are continually updated, such as those provided by public pathogen sequencing projects. Requiring authors to include within their paper a web link to the Microreact containing their trees and metadata provides a standardized approach to data storage and sharing, facilitating the interpretation of genomic datasets beyond the publication and by readers with minimal knowledge of bioinformatics.

## Methods

**Fig. 1.**
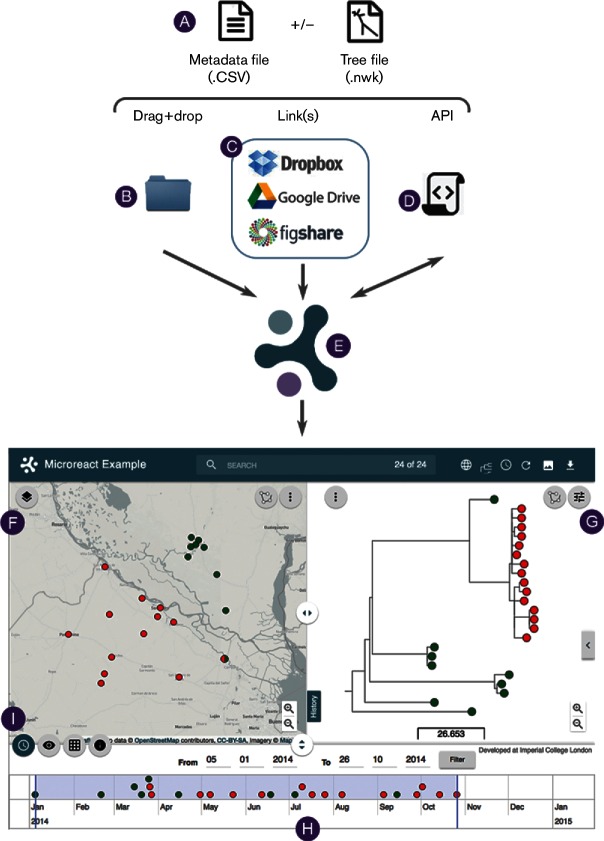
The Microreact workflow: input files (A); upload options (B, C and D); web application (E); map view (F); tree view (G); timeline (H); menu buttons to control bottom panel view (I), from left to right: timeline, view, table, info.

The overall workflow for creating and interacting with a Microreact can be seen in Fig. 1. Briefly, a data file containing user-defined variables along with geographical and/or temporal data is required, and a tree file can also be provided if available. Colours and shapes for the variables can be specified within the data file, and are displayed once a Microreact is created. Following upload, the main interface consists of three interlinked components, a map, a tree view and a timeline. A data table can be accessed via an icon within the application. The unique uniform resource locator (URL) assigned to each Microreact can be used to share it with collaborators, and the data used to create it are available to download.

Firstly, we detail data specification, then how to create a Microreact and interact with the data, and finally how to amend the initial view and programmatically link to data via an Application Programming Interface (API). We explain the process of creating a Microreact containing three data types (tree, geographical and temporal), but it is not necessary to provide all these data types to use Microreact.

### Input data file and tree.

The input to Microreact is a data file in comma separated value format (CSV, A in Fig. 1), along with an optional tree file in Newick format (Felsenstein). The CSV file should contain entries as rows and data variables as columns. The format of the column headers is summarized in Table 1. The name of the entries in the ID column must be unique and exactly match those utilized as leaf labels within the Newick tree file. Geolocations should be specified in the latitude and longitude columns as decimals (WGS84). Temporal data should be formatted into three separate columns: year, month and day. Missing data values for month and day will result in the entry being dated to the 1st of January of the year specified. Likewise, missing day values will result on the entry to be dated to the 1st of the month and year specified.

**Table 1.** Column headers of the data (CSV) file

Column header	Value	Example
**Mandatory columns**		
ID	User defined Note: must match leaf labels within the tree file	O157:H7, 1054_5#29
**Optional columns**		
Latitude	Decimal latitude (WGS84)	52.07853
Longitude	Decimal longitude (WGS84)	0.18613
Year	Integer	2016
Month	Integer	02
Day	Integer	15
ColumnName__shape	User defined; options – circle (default), square, triangle, star	Circle (default), square, triangle, star
ColumnName__colour	User defined: hexadecimal colours or valid HTML5 colour names	#0000FF, blue
ColumnName__autocolour	Automatically assigns colours to unique values in a column	

The minimal data required is a CSV with a set of IDs and at least one data column. If geographical, temporal or clustering data are included, the Microreact will link them to the data table via a map, timeline or tree, respectively. For all possible combinations of data visualization see Table S1 (available in the online Supplementary Material).

### Specifying colours and shapes within data.

Optional columns can be used to assign shapes and colours to any data variable for display (Table 1). Column Name__shape and ColumnName__colour assign shape and colour to a variable, respectively. Furthermore, the addition of ‘__autocolour' to any column name will automatically assign colours to unique values within a data column. Those variables in columns with neither colour nor shape assignments will only be displayed as leaf labels on the tree.

### Creating a Microreact.

The input CSV and Newick files can be provided to Microreact in three different ways.

(**i**) **Dragging and dropping the files from a local directory onto the Microreact upload page (B in Fig. 1).** The data are checked for consistency to ensure that all the entry identifiers in the ID column are unique, and that for each leaf label in the tree file there is a matching identifier within the ID column in the data file (a warning is displayed if not). The user can specify a project name and description, and a contact email during the upload. Clicking on ‘create project’ uploads data and creates the Microreact URL, which, by default, consists of a random eight alpha-numeric ID (e.g. B1N95p0h) appended to the end of the permanent Microreact URL (e.g. https://microreact.org/project/B1N95p0h).

(**ii**) **Linking to files stored online (e.g. Google Drive, Dropbox, Figshare or ftp; C in Fig. 1).** Links to data files stored on cloud storage servers can be specified during upload. Files are loaded each time a Microreact is opened to reflect any updates to the data in the original files. Full instructions for linking to files on Google Drive, Dropbox and Figshare are provided in the ‘Instructions' section (at http://microreact.org).

(**iii**) **Programmatically via the Microreact API (D in Fig 1; http://microreact.org/api).** The API allows data to be supplied to Microreact via scripts and command line interfaces, and provides flexibility for creating a new Microreact as the direct output from bioinformatic pipelines. A full description of the API is available at the Microreact.org website, and we describe an example within the Results section.

### Interacting with a Microreact.

We have produced a hypothetical dataset that includes a tree, geographical and temporal information. We have provided a screenshot within Fig. 1, but suggest that the reader also browses the data at https://microreact.org/project/B1N95p0h while reading these methods.

The default Microreact window consists of three main views: the map view (F in Fig. 1), the tree view (G in Fig. 1) and the data view (H in Fig. 1). For convenience, the data view contains multiple sections that can be accessed by clicking the icons on the left hand border (I in Fig. 1): a ‘time’ icon for viewing a timeline (default display), a ‘view’ icon for interacting with data variables, a ‘table’ icon for viewing a tabular version of data and an ‘info’ icon showing descriptive user-defined information for a particular Microreact. Each section of the interface can be resized by clicking and dragging the divider icons, or turned on/off using the icons at the top right of the Microreact window. By default, the first variable with defined colours and/or shapes in the CSV is utilized to display coloured icons on the map, tree and timeline. Toggling the visualization between different metadata variables is achieved by clicking the view icon (I in Fig 1). Any variable with a colour/shape defined in the CSV file will have a corresponding button within the ‘Shapes and colours’ section and the ‘Metadata Blocks’ section, and all other variables will have a button in the ‘Labels’ section. Clicking on a button within the Shapes and colours section will update the map/tree/timeline icons with the defined shape/colour. Similarly, clicking on a button within the Labels section will display variables as text labels on the tree only. Finally, in the Metadata Blocks section the user can select variables to be displayed as coloured blocks next to the tree, of particular utility when multiple variables need to be visualized simultaneously.

### Map.

Geographical data are displayed as icons on the map. For variables with a colour/shape definition on the CSV file, the map icon is displayed accordingly. Selecting a single point on the map by clicking on it will open an ‘info window’ listing the data entry (or entries) defined for that location, and will also highlight their corresponding positions on the tree and timeline. The entries in the table view are filtered to reflect only those on the selected map location. Furthermore, for any coloured variable with more than one value at a particular location, the distribution of values is indicated as a pie chart on the map location and on the info window.

### Tree.

The default tree type display is radial and can be navigated using standard click–drag–zoom. Clicking the ‘show controls’ button at the top right of the tree window allows tree shape to be changed to rectangular, circular, diagonal or hierarchical, and both node and label sizes can be changed using the sliders. Further options to hide/show leaf labels and to align leaf labels (for circular, rectangular and hierarchical tree shapes only) are available by right-clicking anywhere within the tree view. Aligning the tree labels is recommended when displaying metadata blocks on the tree.

Clicking on a single leaf on the tree will highlight its position on the map and also the timeline. Clicking on an internal node on the tree will filter both the map and timeline by hiding any data not included within the selection. Clicking anywhere outside of the tree in the window will remove filters. To view a single branch in more or in less detail, right-clicking on an internal node displays a menu with the options to redraw a subtree and to collapse/expand a subtree. Redrawing displays the data selected as a subtree within the entire tree view, and filters both the map and timeline to show only those data corresponding to the subtree. To return to the original tree, the user should right-click anywhere on the tree window and select the ‘redraw original tree’ option. Alternatively, if a number of subtree iterations have taken place, the ‘History’ panel to the left of the tree view records each subtree step and allows a user to choose the view level.

### Timeline.

The timeline displays points for any data containing temporal information. The timeline displays stacked data points and will cluster for larger datasets, but can be zoomed in using standard mouse controls. Individual points can be clicked on the timeline, which highlights the corresponding map and tree points. Initially, all data are displayed and indicated by a bounding box bookended by two vertical blue bars. To visually inspect the impact of temporal data on the appearance of the map and tree, the user may specify a time interval of interest by either dragging the blue bar, or defining specific ‘from’ and ‘to’ dates in the text boxes directly above the timeline. Any data points outside of the defined time interval will be filtered out on the map and on the tree (locations and leaves will disappear, respectively). Furthermore, if the map location was represented by a pie chart, the chart will update to reflect the values included within the specified time interval only.

### Tabular view.

Clicking on the table icon on the data view will switch from timeline to table providing a way to see the raw data as uploaded within the CSV file. Upon data selection or filtering within the map, tree or timeline view, the table is updated accordingly to only show those data within the filter.

### Viewing sub-selections of the data.

Besides single data point selection, multiple data points can be selected within the map, tree and timeline views by holding the ‘cmd’ button on Mac or the ‘ctrl’ button on Windows and clicking on multiple locations, leaves or time points, respectively. In this way, a user can, for example, select leaves on different branches of the tree. Furthermore, on the top right corner of the map and tree views we provide a ‘lasso’ tool that allows a user to select multiple data points by drawing around them, which, in turn, filters out the data not included in the lasso selection from all the views (map, tree, timeline and table).

We also provide a text search box at the top of each Microreact, where a user can enter free text to query either all data, or, by clicking on the magnifying glass in the search box, a specific data column. In this way, only the data points matching the query string are shown on the map, tree, timeline and table, and the remaining data points are filtered out.

A legend to any coloured variable is available to view by clicking on the ‘<’ tab located at the mid-right edge of a Microreact. This will expand a side bar showing a key for the variable being displayed. Clicking on any of the values in the key will highlight data within the map, tree and timeline.

### User accounts and managing Microreact projects.

Creation of a Microreact can be undertaken directly from the web interface simply by adding files as described above. However, any subsequent modifications to a project created in this way, such as changing the project name, will require the creation of a new Microreact, with the concomitant new URL. Nevertheless, at microreact.org we also enable the users to change projects and manage access through user accounts. The management interface (Fig. S1) is available by clicking on the hamburger icon at the top left of the Microreact homepage, or on the Microreact logo at the top left of a project’s webpage. Users can log in using their Google, Twitter or Facebook accounts, and a list of all the projects created while logged in will be available to manage (Fig. S2), allowing project name/description to be amended, data files (Newick and CSV) to be updated and access control defined. Projects can be designated as ‘private', ‘public', or ‘public and listed' (meaning that permission is given to highlight projects on the microreact.org homepage). Users can also delete projects created while logged in, and access basic project statistics such as date created, last modified, last accessed and number of visits. Furthermore, if a project is set as public and listed the user can specify a unique custom project ID (e.g. myfavouriteproject), which replaces the random project ID set by default and is appended to the end of the permanent Microreact URL (e.g. https://microreact.org/project/myfavouriteproject).

### Data download and re-use.

All data (CSV and tree files) used to create a Microreact can be downloaded from the download menu (top right) for re-use. Furthermore, images of the whole Microreact webpage, as well as individual views of the map, tree, timeline and legend can be saved in PNG or SVG (tree only) format, allowing snapshots of the data of particular interest to be saved.

### Customising the initial view.

A number of variables can be appended at the end of a Microreact URL to amend the initial default tree display with preferred values for tree type, tree label text and node size (Table 2). The initial variable must be appended following a ‘?’ character. Each variable must be specified with ‘=’ and separated by ‘&’. For example, for our demonstration visualization (https://microreact.org/project/B1N95p0h), to set the initial tree type as circular, turn labels off and increase node size to 50 we can use the following: https://microreact.org/project/B1N95p0h?tt=cr&tl=0&tns=50.

**Table 2.** Tree formatting options to be specified in the Microreact URL

Variable	Code	Expected values	Format
Tree type	tt	rd	Radial (default)
		rc	Rectangular
		cr	Circular
		dg	Diagonal
		hr	Hierarchical
Tree labels	tl	0	Labels off
		1	Labels on (default)
Tree node size	tns	1 to 200	–
Tree text size	tts	1 to 200	–

### Technical specification.

Microreact is a React (http://facebook.github.io/react/) and Node.js (http://nodejs.org) application written in JavaScript, HTML and CSS. Geographical data are represented using the Leaflet JavaScript library (http://leafletjs.com) with tiles from the Mapbox mapping platform (https://www.mapbox.com), tree visualization is provided by the Phylocanvas JavaScript library (http://phylocanvas.net) and timelines are visualized using vis.js (http://visjs.org). User authentication utilizes Passport (http://passportjs.org) and layout utilizes Material Design Lite Components (http://getmdl.io). Project data at microreact.org are stored in a MongoDB database. Microreact is freely available at www.microreact.org and runs within any modern web browser.

## Results

### Microreact applied to infectious-disease epidemiology

To demonstrate the use of Microreact within genomic epidemiology, we created a small hypothetical dataset, based on the clustering of 24 pathogen genomes with associated data including location, date of isolation and antibiotic susceptibility. We applied the ‘__colour' option (Table 1) to the antibiotic susceptibility variable in the CSV file to specify sensitive (green) or resistant (red). A location where both a sensitive and a resistant isolate were found is indicated automatically by a pie chart on the map. The initial view can be seen in Fig. 1 and also at https://microreact.org/project/B1N95p0h, where all data files (CSV and Newick) are available to download. Within our hypothetical dataset all the resistant samples group into one tight cluster on the tree (G in Fig. 1), as indicated by the node with red leaves, and are also geographically located within the same region (F in Fig. 1). By changing the tree type from ‘radial’ to ‘rectangular’ we can investigate the fine-scale clustering within this branch in more detail. The antibiotic phenotype is displayed on the timeline (H in Fig. 1) and informs about the approximate time of emergence of resistance within our sample. By dragging the right-hand timeline bar all the way to the left, we can then gradually move the bar to the right to ‘replay’ the potential chain of events and explore the relationships between the temporal and spatial spread of data.

### Examples of published local and global transmission events visualized within Microreact

**Fig. 2.**
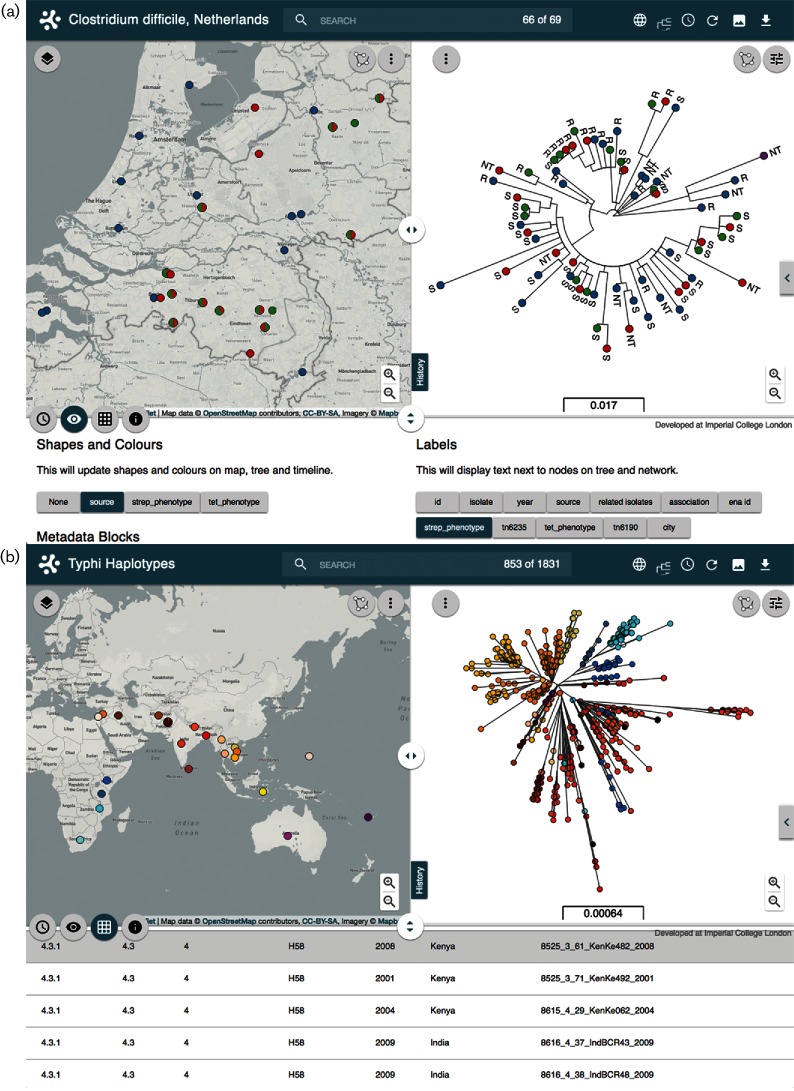
Visualization of local and global transmission events on Microreact. (a) Local, intra-species transmission of *C. difficile* visualized as closely related isolates from different sources sharing location and antibiotic phenotype. The bottom view panel shows the source selected as a colour filter for map and tree, and the streptomycin phenotype selected as a tree label. (b) Inter-continental transmission of *Salmonella* *enterica* serovar Typhi evidenced by the presence of closely related strains from Asia and Africa within the H58 clade. The country was selected as a colour filter for the map and tree, and the bottom table panel shows examples of closely related samples from Kenya and India.

We created two Microreacts to visualize previously published data that have indicated local, inter-species transmission of *Clostridium difficile* (Fig. 2a), and of global, inter-continental transmission of *Salmonella enterica* serovar Typhi (Fig. 2b), respectively. Knetsch and colleagues employed WGS to investigate the relatedness of *C. difficile* isolates from humans and animals in Dutch pig farms (Knetsch *et al.*, 2014). They reported several cases where farmers and pigs were colonized with identical or very similar isolates, which also shared the same antibiotic-resistance determinants. These observations represented evidence of inter-species transmission. For the visualization of these data on Microreact (https://microreact.org/project/rJHFIV-a), we applied the same colour scheme chosen by the authors for the source of the strains, clinical (blue), farmer (green), pig (red) and reference (purple). In addition, Microreact automatically presents pie charts on the map for the locations of the 11 farms where strains were isolated from both human carriers and pigs (Fig. 2a). Importantly, the combination of the information presented as separate tables and figures in the original publication, and the use of coloured metadata variables on the map, tree and timeline, provide not only a clear visualization of the potential inter-species transmission events reported by the authors, but also a user experience for the exploration of data. Moreover, all data can be downloaded for further utility.

Wong and co-workers have recently shown that the H58 multi-drug-resistant haplotype of *Salmonella enterica* serovar Typhi has likely transferred from South Asia to South-East Asia, Western Asia, East Africa and Fiji (Wong *et al.*, 2015). These candidate transfer events can be visualized in Microreact by applying a colour to the different haplotypes and countries where the strains were isolated (http://microreact.org/project/styphi). Initially, by default, the ‘clade’ data are shown within the map, tree and timeline with the H58 haplotype (clade 4.3.1) coloured red and representing the largest clade on the tree. The pie charts on the map represent the distribution of haplotype by country, indicating the prevalence of H58 in the Indian subcontinent, South-East Asia and East Africa. By selecting and redrawing the subtree of the haplotype H58 cluster, the map view updates with the geographical distribution of H58 strains only (Fig. 2b). By then changing the colour filter to indicate country of origin, the two candidate transmission events reported by the authors are easily visualized as clusters containing strains from Kenya and India. Selecting Kenya by either clicking on the map location, clicking on Kenya within the legend, or typing the country name in the search box, highlights isolates from Kenya and their close relationships on the tree to isolates from India.

### Linking to dynamic datasets using Microreact

The examples above show two datasets uploaded to Microreact from scientific articles that represent a snapshot of the populations at the time the studies were published, with static data and tree files. However, continuously growing studies require flexible, dynamic ways to add further data. To address this limitation, we provide two alternative ways to produce a Microreact that links to data files hosted on remote servers. Firstly, data can be linked by specifying URLs as input when creating a Microreact (see Methods, and the instructions at http://microreact.org). This can be used to, for example, link to files on Dropbox, Google Drive and Figshare. Once created, any updates to the files hosted at these locations are reflected within the Microreact when the project is re-loaded; thus, maintaining a single Microreact URL that provides the latest data. Secondly, we have a comprehensive API that allows scripting to link and produce a Microreact programmatically. The API provides functions for creating and parsing data files prior to visualization and for defining data display parameters. In this way, we can link to data that may not adhere to the standard format required by Microreact. To demonstrate this functionality we have created a Microreact that links and displays up-to-date data from the ongoing National Center for Biotechnology Information (NCBI) Pathogen Detection Project (http://www.ncbi.nlm.nih.gov/pathogens/). This initiative provides open access bacterial genome sequences and the output from bioinformatic pipelines from various sources (food, agriculture, environmental sources and patients) to aid public-health scientists investigate disease outbreaks. For a number of pathogenic species, genome sequences are clustered to identify relationships and results are provided via anonymous ftp in a single folder containing the latest analyses (updated daily if new data are submitted).

**Fig. 3.**
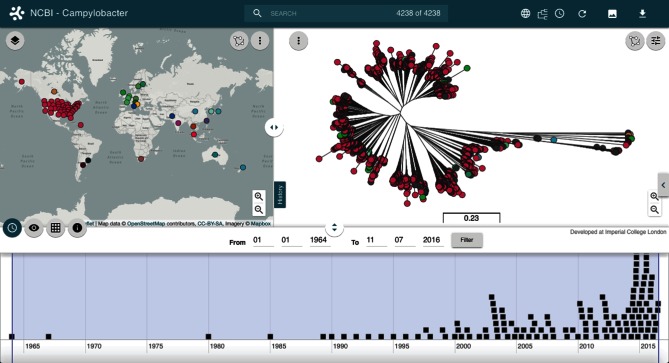
Visualization of *Campylobacter* data from the NCBI Pathogen Detection Project linked live via the Microreact API. The data points are automatically grouped on the timeline. The visualization reflects the 4238 *Campylobacter* spp. genomes available as of 16th November 2016.

The output results include a tree file (Newick) and a data file, which can be used to create a Microreact. Using the API we can programmatically specify which fields in the data file should be used within the Microreact and also add additional columns if needed. For example, data at the ftp site does not contain latitude and longitude values for geographical display. Using the Microreact API we can create a script (see https://microreact.org/tutorials/ncbi) to automatically geocode the contents of a specific column (geo_loc_name) containing location information (country level or USA state), define a colour for each country and provide the data to display on the map. At the same time the script can split the date field (collection_date) into day, month and year columns for use on the timeline. Results from the *Campylobacter* sp. data can be seen in Fig. 3 and at https://microreact.org/project/ncbi-campylobacter?tl=0. Every time the visualization is loaded, Microreact connects to the NCBI server via the API, the latest versions of the data and tree files are parsed and loaded, and data points within the timeline are clustered to optimize display (zoom to expand). An important application, in particular for public-health traceback investigations and outbreak response, is that the most recent samples can be quickly highlighted via the timeline, pointing to their location on the tree and on the map. In this way, incoming genome sequences from a recent outbreak can be swiftly placed in the context of the population, and the samples most closely related to the new genomes can be identified, including their locations and times of isolations.

## Discussion

Microreact is a user-friendly and flexible web-application for creating and sharing interactive visualizations of population genomics data linked to geographical, temporal and associated metadata. Data are presented within Microreact with interactive maps, trees and timelines (F–H in Fig. 1), allowing flexible exploration and querying by audiences with differing expertise. Data are available for download, and can be shared via a permanent URL for collective interpretation or hyperlinked within publications.

The applications of Microreact extend beyond the field of phylogenomics, as any combination of geo- or temporally-referenced data can be used to create a Microreact visualization, without the need of a tree (Table S1). Nevertheless, the genetic information within a species or a population may also be geo- and temporally-referenced, as variation across species and populations is a function of both their environment (space) and their evolutionary history (time). Thus, Microreact provides a simple method to visualize the genetic variation referenced to space and time, which is important to understand both the long-scale, global evolution of an organism, as well as the short-scale, local evolution of a population. For example, since its release in June 2015, Microreact has been used and included in publications on the study of post-vaccine evolution of *Streptococcus pneumoniae* (Croucher *et al.*, 2015), of gene flow of *Yersinia enterocolitica* (Reuter *et al.*, 2015) and within the Ebola outbreak response (Gardy *et al.*, 2015). While we focus on the area of microbial genomics and epidemiology, Microreact can be applied to the study of other organisms. For example, a recent human phylogeny based on the Y-chromosome sequence (Hallast *et al.*, 2015) is showcased on the Microreact website (http://microreact.org/project/NyqrhlsB).

A number of innovative features make Microreact particularly suitable for pathogen surveillance, as well as for outbreak and epidemiological investigations, where the variation in genomes allied with temporal and locational data is utilized to attempt to understand the transmission routes of a pathogen, the spread of high-risk clones, and the emergence of antimicrobial resistance. Firstly, by linking to data on remote servers via ftp or programmatically via a flexible API, Microreact can provide a method for creating automatic visualizations of dynamic datasets that are regularly updated, such as the Pathogen Detection Project at the NCBI (Fig. 3). It is worth noting that the automation of visualization in this way calls for better practices of metadata storage and standardization for large projects. In our example, the location data consists of country information, input using either full name (e.g. Vietnam) or a three-letter code (e.g. USA). Adopting accepted standard codes like the ISO 3166 International Standard codes for countries and their subdivisions (http://www.iso.org/iso/home/standards/country_codes.htm) would minimize the need for data parsing, as well as reducing errors or inconsistencies. Secondly, the visualization of static or dynamic datasets can be made available through an easy to use, shareable interface, which provides a flexible means of collective data exploration and interpretation by groups of end-users (e.g. laboratory scientists, epidemiologists and public-health workers). Lastly, if antibiograms are included in the routine surveillance, drug susceptibility data can be visualized on Microreact as data blocks next to the tree, and as pie charts on the map, facilitating the examination of antimicrobial resistance.

As the number of genome sequences continues to increase in public archives, so does our understanding of the importance of annotating them with accurate metadata for location, date of isolation, host, etc. While significant resources have been directed towards making sequence data open access (Cochrane *et al.*, 2016; Gibson *et al.*, 2016), the same does not necessarily apply to metadata and phylogenetic trees, where the main data source often remains unlinked within the individual publications. This poses a number of issues for data access: (i) not all scientific journals are open access; (ii) a static image of a tree is usually presented for publication, but the tree file is not; thus, hindering any further exploration of the results by the community; and (iii) there is no consensus on the format of metadata tables, not even for the same organism. Using specific examples we have shown how Microreact can integrate the information usually published separately as static figures and tables into one fully interactive visualization with tree, map, timeline and table (Fig. 2a, b). The ability to share, download and reuse such data addresses a number of these issues. In addition, user accounts and project management allow a user to customize and share the Microreact prior to and during publication.

While we provide methods for visualizing trees in conjunction with maps and timelines, there are additional visualization methods that could aid the interpretation of linked data. As datasets become larger and trees increase in size, visualizing a tree with thousands of leaves becomes a scalability issue, and efforts are needed to provide either alternative visualizations and/or automatic collapsing of branches. Much in the same way that data points can be clustered on a map and timeline to provide an initial summary (e.g. at country level), high-level clustering of tree branches with subsequent expansion of nodes of interest may offer a solution. Alternative visualizations may also add value to the data on a Microreact. For example, within infectious-disease epidemiology, networks are often used to visualize potential transmission paths, and are likely to add useful information in conjunction with the map and the timeline. Similarly, graphical summaries of data such as pie and bar charts are often useful, either allied to specific branches on the trees, or for simple overviews of metadata. We are further developing Microreact to allow such visualizations to be included.

In summary, Microreact enables rapid, open introspection and correlation of clustering, geographical and temporal data in the distributable form of a web link. All files (data and tree) can be downloaded from Microreact and used in other analysis packages. Microreact provides both a richer visualization experience and better access to datasets to a broader usership, contributing to transparency and reproducibility in research, as well as increasing the utility and impact of research results. We encourage scientific journals to provide access to population genomic data interpretation by the provision within publications of a URL to software such as Microreact that is pre-loaded with the genomic analysis results and associated data.

## Supplementary Data

710 Supplementary File 1
