# Supplementary material for: Microreact: visualizing and sharing data for genomic epidemiology and phylogeography
Source: Microb Genom. 2016 Nov 30;2(11):e000093. doi: 10.1099/mgen.0.000093 (PMC5320705; doi:10.1099/mgen.0.000093)
Supplement: Supplementary File 1 [file mgen-02-93-s001.docx]

SUPPLEMENTARY DATA

1) Logging in and Project Management


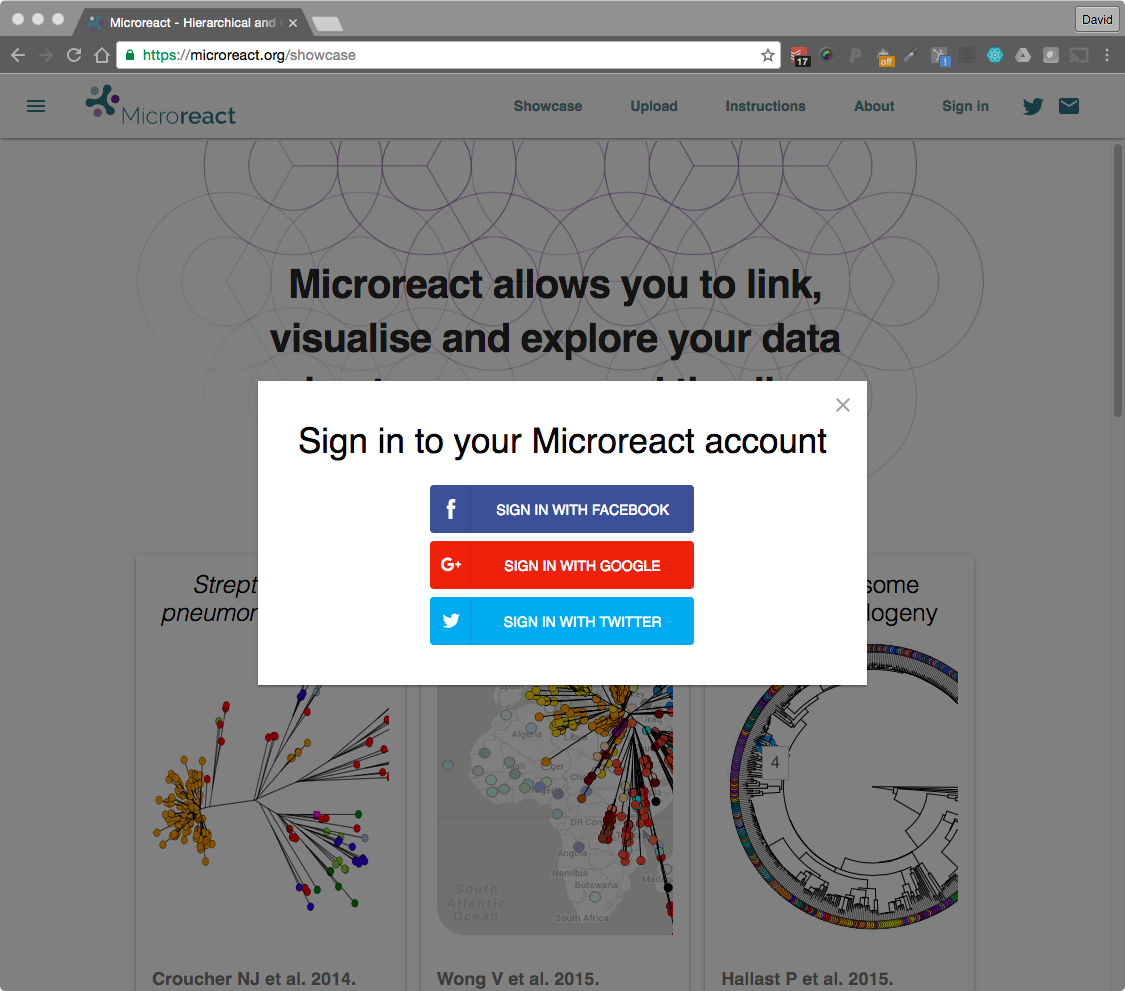


**Figure S1.** Clicking ‘Sign In’ at the top right of the homepage allows users to choose and enter their login details.


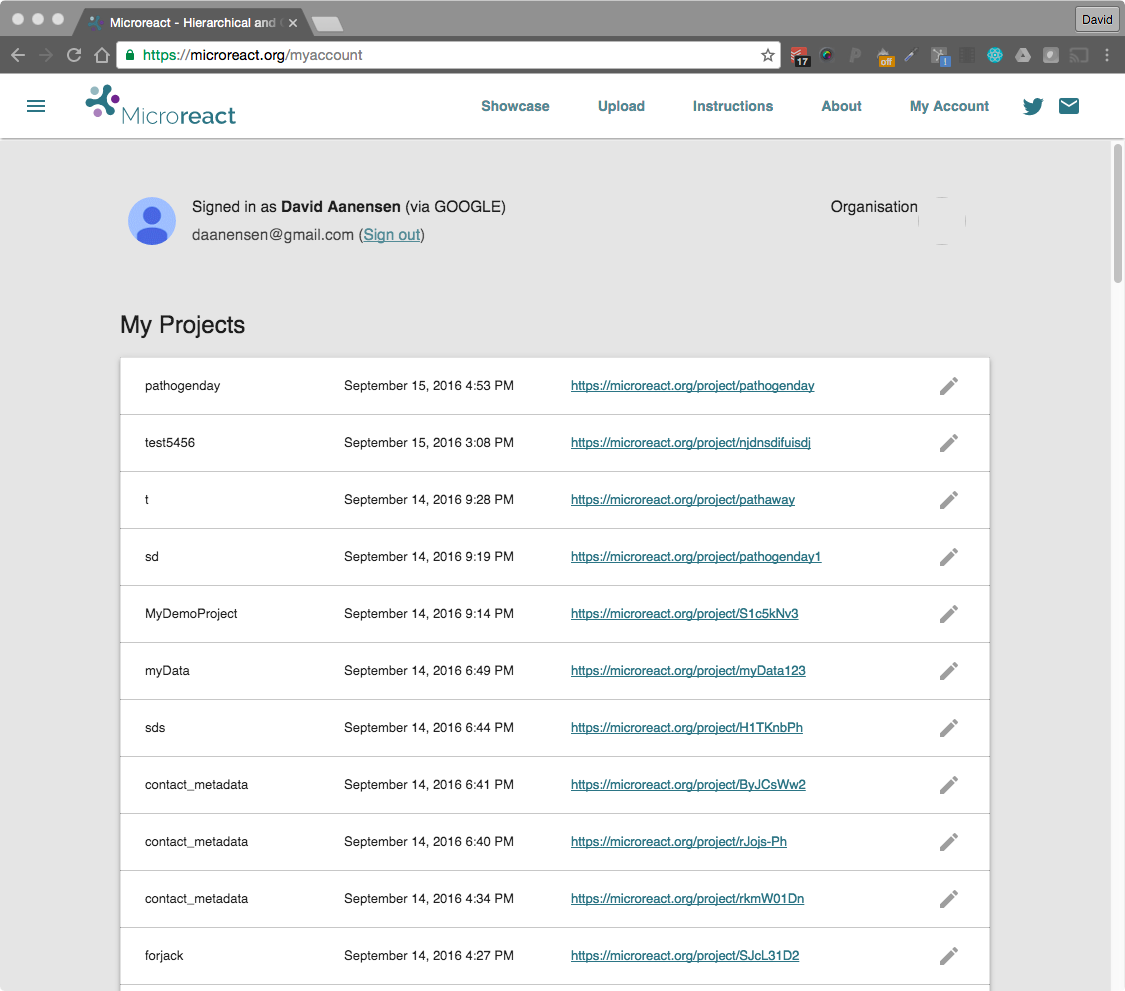


**Figure S2.** The ‘My Account’ window lists all projects created by a particular user. Clicking on the ‘edit’ icon to the right of a project brings up the management window.


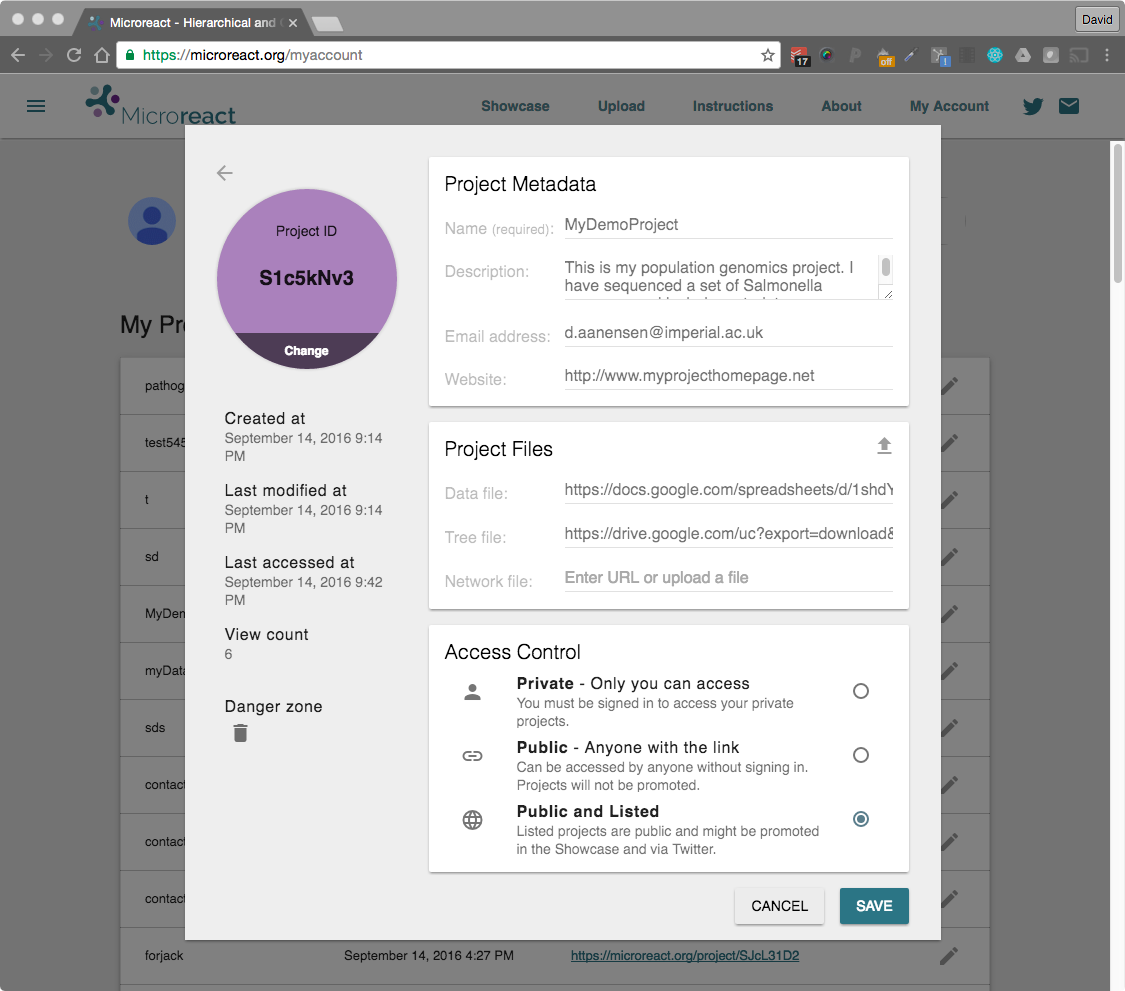


**Figure S3.** The project management window.

For a particular project, the metadata can be amended within a specific linked file, or entire project files can be added or replaced. Access Control allows a user to define a particular Microreact as either:

- Private: A User must be signed in to access private projects.
- Public: Can be accessed by anyone with the URL, without signing in.
- Public and Listed: Listed projects are public and might be promoted and listed in the Microreact.org showcase section and via Twitter.

Basic Statistics about a particular Microreact are available: Date created, last modified and last accessed. ‘View count’ the number of times the Microreact has been visited.

The ‘Danger zone’ contains a dustbin icon that can be clicked to delete a Microreact – the user will be prompted for final confirmation before full delete.

Changing the Project ID: If a Microreact is ‘public and listed’ the user may click the ‘change’ button at the top left and specify a custom ID which will be used in the Microreact URL as a convenient link to the project.

| Input Data | | | | | Microreact will contain | | | |
| --- | --- | --- | --- | --- | --- | --- | --- | --- |
| CSV | | | | Tree |  |  |  |  |
| id | metadata columns | Lat\|lon |  |  | Table | Map |  |  |
| id | metadata columns |  | Day\|month\|year |  | Table |  | Timeline |  |
| id | metadata columns | Lat\|lon | Day\|month\|year |  | Table | Map | Timeline |  |
| id | metadata columns | Lat\|lon |  | Nwk | Table | Map |  | Tree |
| id | metadata columns |  | Day\|month\|year | Nwk | Table |  | Timeline | Tree |
| id | metadata columns | Lat\|lon | Day\|month\|year | Nwk | Table | Map | Timeline | Tree |

**Table S1.** A Microreact can be created with different combinations of data. The different combinations of input and the resulting Microreact views are indicated.
